# Primary care patients’ and providers’ perspectives about an online weight management program integrated with population health management: Post-intervention qualitative results from the PROPS study

**DOI:** 10.1016/j.pecinn.2022.100057

**Published:** 2022-06-11

**Authors:** Ronen Rozenblum, Barbara A. De La Cruz, Nyryan V. Nolido, Saffiya McNulty, Katherine D. McManus, Florencia Halperin, Jason P. Block, David W. Bates, Heather J. Baer

**Affiliations:** a Brigham and Women’s Hospital, Boston, MA, USA; b Harvard Medical School, Boston, MA, USA; c Harvard T.H. Chan School of Public Health, Boston, MA, USA; d Harvard Pilgrim Health Care Institute, Boston, MA, USA

**Keywords:** Weight management, Overweight, Obesity, Primary care, Online program, Population health management

## Abstract

**Objective:**

To assess patients’ and providers’ attitudes about the online weight management program and population health management approach in the PROPS Study, which examined the effectiveness of these strategies in primary care.

**Methods:**

We conducted semi-structured interviews with 22 patients and nine providers. Using thematic analysis, we analyzed transcripts of the interviews to identify key themes.

**Results:**

Most patients found the online program to be well-structured and easy to use, although a few noted that the information was overwhelming or could be more personalized. Patients mentioned that the support from the population health managers was critical for their success, and several reported that they would have liked more involvement from their primary care provider or a dietitian. Providers also were satisfied with the interventions, and several stated that the population health management support was helpful because it added accountability. Providers suggested that the interventions could be improved by tailoring the information and integrating the online program with the electronic health record.

**Conclusion:**

Most patients and providers were satisfied with the interventions, with several recommendations for improvements.

**Innovation:**

These findings give additional information about patients’ and providers’ experience with this innovative approach for managing overweight and obesity in primary care.

## Highlights

• Patients and providers were satisfied with the online weight management program.
• The population health management support was critical to patients’ success.
• Weight management programs should be tailored to patients’ needs.
• Online weight management programs should be implemented more broadly.

## 1 Introduction

Primary care providers (PCPs) play an important role in assisting patients with weight management [[1], [2], [3]]. However, PCPs often do not counsel patients about weight for a variety of reasons, such as insufficient time or lack of adequate training [[4], [5], [6], [7], [8]].

Evidence from many studies, including several in the primary care setting, indicates that online weight management programs can help people achieve and maintain clinically meaningful weight loss [[9], [10], [11], [12], [13], [14], [15], [16], [17], [18], [19], [20]], and patients and healthcare providers have expressed positive opinions about these programs [21]. Despite these findings, online programs are not being widely implemented in primary care, and it is unclear whether they are effective or scalable in real clinical practice [14,21].

To address this, we conducted the Partnerships for Reducing Overweight and Obesity with Patient-Centered Strategies (PROPS) Study, which was a cluster-randomized trial designed to compare the effectiveness of a combined intervention, including an online weight management program plus population health management support, with the online program only and with usual care. The online program (BMIQ, Intellihealth Inc) had patient and professional interfaces. The patient interface included educational sessions about nutrition and behavior change, exchange-based meal plans and sample menus (as well as a calorie goal), and tools for tracking weight, diet, and physical activity. The professional interface included patient monitoring and alerts, progress notes, and reporting features. Patients in the online program only group were registered for the online program and sent instructions about how to use it; they also were offered a brief phone consultation with a registered dietitian. Patients in the combined intervention group received the same components as the online only group plus additional support from a population health manager (PHM), a nonclinical staff member who works with the primary care practices. The role of the PHMs was to monitor patients’ progress in the online program and to conduct outreach according to a specific protocol. The intervention period was 12 months, and patients were followed for 18 months [22].

A total of 840 patients enrolled in the study. There were significant differences in weight change and other weight-related outcomes by group. Participants in the combined intervention group had the greatest weight loss at 12 months, followed by participants in the online program only group and then by participants in the usual care group [23]. The intervention, methods, and main results from the trial have been described in detail elsewhere [22,23].

At the end of the trial, we also collected qualitative data from patients and providers who were involved in the study, to help better understand and evaluate the interventions. Specifically, the purpose of this qualitative sub-study was to assess patients’ and providers’ attitudes towards and experience with the online program and the population health management approach. This article describes the methods and results from the qualitative data.

## 2 Methods

### 2.1 Overview of design

To obtain more detailed feedback about the interventions in the PROPS Study, including the online program alone or combined with additional support and outreach from PHMs, we conducted semi-structured interviews with 22 enrolled patients and nine providers between June and December of 2018. This qualitative study took place at Brigham and Women’s Hospital (BWH) in Boston, Massachusetts, and was reviewed and approved by the Partners HealthCare’s Human Research Institutional Review Board (protocol # 2015P002372). The trial also was registered on ClinicalTrials.gov (Identifier: NCT02656693).

### 2.2 Interviews with patients

In the interviews, we sought to obtain feedback from participants about their experience and satisfaction with the online program, the additional support and outreach from PHMs for those in the combined intervention, and related challenges and opportunities for improvement. For this reason, we employed purposive sampling to recruit participants. We only enrolled patients who were assigned to the online program alone (OP) or combined intervention (CI) groups and who had completed the 18-month follow-up period for the trial were eligible to participate in the interviews. Patients completed a final survey at 18 months, which included a multiple-choice question to assess their interest in participating in a discussion about their experience in the study. Research staff sent a recruitment letter and information sheet and followed up with a phone call one week later to describe the interview and confirm participation. Scheduled participants provided verbal informed consent and were interviewed by a trained member of the research team (RR, BD) in either English or Spanish, depending on language preference. Patients who completed the interview received a check for $50.

### 2.3 Interviews with providers

We conducted semi-structured interviews with providers to gather their perspectives about the impact of the interventions (online program alone or with additional support and outreach from PHMs) on the care they provided to patients, positive and negative aspects of the intervention, and recommendations for improvement. We recruited providers who had been involved in the PROPS Study in different roles (primary care physicians, PHMs, and registered dietitians). For primary care physicians, we recruited those who had at least six enrolled patients in the OP or CI groups of the trial. The Principal Investigator (HJB) contacted providers by email to describe the interviews, assess interest, and address any questions. Five physicians, three PHMs, and one registered dietitian (the Director of Nutrition at BWH) provided verbal informed consent and were interviewed by an experienced member of the research team (RR). Providers who completed the interview received a check for $50.

### 2.4 Patient and provider interview guides

Semi-structured patient and provider interview guides (see Appendix A & B in Supplementary Material) were developed by the research team to facilitate discussion and standardize data collection. The interview guides were developed based on a literature review and expert opinion. The guides were then piloted with several content experts, including our Patient and Stakeholder Advisory Committee (PSAC) members, to ensure clarity and appropriate length. The patient interview guide asked questions about patients’ activities and experience with the online program and the population health management support (if applicable), perceived value, main weaknesses and/or challenges, and suggestions for improvement. The provider interview guide included a few sections that focused on providers’ involvement in the PROPS study, their attitudes towards the interventions (online program only or with additional support from PHMs), and their willingness to use the program in the future.

### 2.5 Data collection and analysis

All interviews lasted between 15 and 30 minutes and were conducted by phone. Interviews were recorded and transcribed with the consent of the participants, and identifying information was removed during transcription. Using a thematic analysis approach, three authors (RR, BD, SM) systematically coded the transcripts to identify, categorize, and sort key concepts and codes. Codes were then grouped into emergent themes and relationships after iterative review and discussion. In cases where there was disagreement, a fourth author (HJB) assisted in reaching consensus.

## 3 Results

### 3.1 Patients’ perspectives

#### 3.1.1 Patient characteristics

We approached 33 patients who had expressed interest in doing the interviews. Of those, we conducted interviews with 22 patients who were enrolled in the OP or CI groups, and we reached saturation of themes. Of the 22 patients who completed interviews, 10 were in the OP arm and 12 were in the CI arm (see Table 1); 54.5% were female, 90.9% were white and English-speaking, 77.3% had a college or advanced degree, 27.3% had type 2 diabetes, and 100% had hypertension. At enrollment, mean age was 61.8 years, mean weight was 94.5 kg, and mean BMI was 32.3 kg/m^2^. Their mean weight loss at 12 months (the primary outcome of the trial) was 6.4 kg.

**Table 1:** Characteristics of patient participants (n=22)

Characteristic	
Age, years, mean (std)	61.8 (6.4)
Intervention group, n (%)	
Online program (OP)	10 (45.5)
Combined intervention (CI)	12 (54.5)
Gender, n (%)	
Male	10 (45.5)
Female	12 (54.5)
Race / ethnicity, n (%)	
White, non-Hispanic	20 (90.9)
Hispanic	1 (4.6)
Other	1 (4.6)
Primary language, n (%)	
English	20 (90.9)
Spanish	2 (9.1)
Education, n (%)	
High school graduate or less	1 (4.6)
Some college	3 (13.6)
4-year college graduate	7 (31.8)
More than 4-year degree	10 (45.5)
Missing	1 (4.6)
Self-reported health status, n (%)	
Poor or fair	2 (9.1)
Good	12 (54.6)
Very good or excellent	7 (31.8)
Missing	1 (4.6)

#### 3.1.2 Patient engagement and experience with the online program

Most patients reported using a single device to access the online program, which included a desktop computer, laptop, tablet, or smartphone. Although most of them used the online program frequently (e.g., twice or more per day or a couple of times per week), a few used it more at the beginning of the study and gradually decreased their usage over time. Below are representative comments from two participants:

*I tended to log after breakfast in the morning, and then at nighttime I would catch up with putting in the other two meals. I was pretty dedicated. I did it almost every day. It might have been once or twice or five times over the course of the 18 months where I might have fell behind a couple days, but I always had it.*

*I’ll tell you, during the period, the 18-month period, I was relatively religious about going on, and being very conscientious of watching the videos, and having all of my meals on there. I was very committed to it. Recently, because I’m not on it that much, I feel like I’m at a plateau. I haven’t been going on.*

The majority of participants were very satisfied with the online program. They found it useful, well-structured, and simple to use. Some of the most important themes related to their positive attitudes and experience with the online program pertained to information/resources and time/efficiency. Specifically, they enjoyed using the online program because it helped them manage their weight and made them more mindful about their diet and health. For example, one patient stated that:

*“To be completely honest, it surprised me because I thought at my age I would never lose any more weight, yet I lost a hell of a lot more weight than I ever thought I would be able to. I'm extremely happy with that.”* Additional comments and quotes from patients are included in Table 2.

**Table 2:** Patients’ attitudes about the online program and recommendations for improvement.

Representative Comments, Quotes, and Themes Related to the Online Program
• “I loved it. I thought it was fantastic. It was the kind of kick in the pants that I really needed. Not difficult, there were very few challenges. I liked the whole format of both the online link with Lose It!, and with the program, and the combination of the quick videos.” (Themes: information/resources, time/efficiency) • “I'm a professional weight-loser over the last 50 years, but I think what I found in the BMIQ was that your information there was pretty much state of the art about balanced diet. I kinda followed it as close as I could, and I felt very satisfied in the foods I was eating. Generally, I thought it was very up-to-date and accurate information for resources.” (Theme: information/resources) • “It was an easy list, if you will, because there wasn’t a lot of commitment. I do like the fact that, unlike Weight Watchers or other programs, it’s not as time consuming in the flesh. You don’t have to go to meetings. I could do it on my own terms. That part of it was really great.” (Theme: time/efficiency)
Recommendations for Improvement
• Address the specific needs of participants. To improve, consider personalizing certain BMIQ features, such as electronic reminders, videos, and menus. Menu planning, stress management, and psychological aspects of nutrition were suggested topics for additional resources. (Theme: personalization) • Help participants utilize the online tools effectively. Participants found that there was too much information to review, choose, and track. To improve, prioritize the importance of tasks. (Themes: information/resources, time/efficiency) • Improve integration of the BMIQ program with the LoseIt! mobile app. As one participant stated, “I would say the more you could put into the mobile experience, I think the better stickiness you'll have with people who are using it." (Theme: integration) • Improve the shared experience of using the program by allowing participants to communicate with each other. Allow for participants’ program activity to be shared with their providers at visits. (Theme: communication)

While most patients valued the entire program, a couple of participants only saw the value in certain features. For example, one patient stated that:

*Other than recording my weight every day, I didn't have any other use for it. It didn't give me any information I needed or could apply.*

A few participants also expressed dissatisfaction and challenges, citing timing and lack of availability as reasons why they were unable to use the system. Below are representative comments from two participants who described issues with the program:

*For me, it was confusing. There was a lot of information, a lot of reading, a lot of time. It was time consuming.*

*I felt like I missed the boat. I would add that there wasn't an accountability piece for me... I was too overwhelmed.*

When asked for suggestions or recommendations to improve the online program, some participants mentioned that their physicians could have been more involved with the intervention (e.g., discuss their weight loss progress during visits). Other patients suggested that certain features of the program should be more personalized, such as electronic reminders, videos, and menus. A few participants suggested adding other resources (e.g., menu planning, stress management, and psychological aspects of nutrition) and improving the shared experience of using the program by allowing participants to communicate with each other. Consistent with this notion, some of the most important themes related to their suggestions or recommendations to improve the online program included personalization, information/resources, time/efficiency, integration, and communication. Representative comments and quotes from patients are included in Table 2.

Finally, most patients stated that they would like to continue to use the online program in the future and had already recommended it to friends and family members. Others stated that they would continue using only certain features or that they would not use the program again, preferring in-person programs.

#### 3.1.3 Patient attitudes about the support and outreach from population health managers

Patients in the combined intervention group felt strongly that the support and outreach from the PHMs were critical for the success of the intervention. Specifically, they saw great value in having human support and outreach as part of the program, rather than only receiving the online program. Representative comments were:

*I did find it useful. It was kinda reassuring to have somebody checking on me and helping me—remind me that I was on this journey.*

*I think it was important to know that there was more than just a computer on the other end.*

*I enjoyed talking to a person once in a while. I think that it's useful to talk to somebody, even if the person doesn't have any sort of amazing insights that you wouldn't have otherwise. From watching the video, just somebody there checking up and encouraging you, saying that you're doing really well or that you could do better or who knows what. It's good.*

*It was nice to know that there's someone tracking it and was interested in knowing about the process. And I found [PHM’s name] sympathetic and willing to listen to what I had to say about what was working and what wasn't. So, I do think the support element was an important part of the study, and I appreciated it.*

A few patients expressed that the support they received was inconsistent and not aligned with their goals. For example, one patient stated that:

*I think they're good to have. I like the concept of it. It was maybe a little too infrequent and maybe also a little too general for me to really equate it with a high level of value.*

Other participants appreciated the support from the PHMs but would have also liked more involvement from a dietitian or their physician as part of the intervention.

### 3.2 Providers’ perspectives

#### 3.2.1 Providers’ role and involvement in the PROPS Study

We conducted nine interviews with providers (i.e., physicians, PHMs, and dietitian) who were involved in the study, and several themes emerged in alignment with the interview guide. Of the nine providers, two had patients enrolled in the OP group, six had patients enrolled in the CI group, and one (the dietitian) had patients in both groups. Most providers felt that they were engaged with the PROPS Study. They stated that they received information about the status of their patients while in the study, received notifications via the electronic health record (EHR), and heard from patients directly during visits. One physician said:

*I asked patients how it was going whenever I saw them and I knew that they were involved in the study. I would periodically get updates through the electronic medical record about what their status was with regards to their weight loss or progress.*

When asked if they talked to patients about their participation in the study, some providers stated that they discussed their experience, progress, and difficulties related to the intervention with their patients. Others stated that they talked only briefly about the study during recruitment or not at all.

#### 3.2.2 Provider attitudes about the PROPS Study

All providers felt that the study helped them provide better care for their patients. Specifically, they stated that the study helped their patients manage their overall health and presented them with a new resource for weight management. When asked to give examples about the impact of the study, providers shared a few success stories about patients who lost a significant amount of weight or were even able to stop blood pressure medications. For example, one physician said:

*One patient stood out as doing remarkably well. He maintained his weight loss for at least a year.*

#### 3.2.3 Provider attitudes about the online program

Although most providers had positive attitudes about the online program, some felt that because of their minimal involvement, they did not know enough about the program to give their perspectives. Consistent with this finding, those who were less familiar and involved with the study could not provide information about the impact of the program on the health and behaviors of their patients. Providers who were more involved and aware of the online program were very satisfied and felt that it had helped their patients manage their weight. Some of the most important themes related to their positive attitudes about the online program included information/resources and accountability. Some representative comments and quotes from providers are included in Table 3.

**Table 3:** Providers’ attitudes about the online program and recommendations for improvement.

Representative Comments, Quotes, and Themes Related to the Online Program
• “It helped me. I think it was another tool to provide the patient in efforts to try to take off weight.” (Theme: information/resources) • “It helped me when I was calling them to check in once a month. I would also check in with them in regards to their diabetes.” (Theme: accountability) • “I did like being able to offer something to patients who were struggling with weight loss because it's so hard for them.” (Theme: information/resources) • “I think it was better care for them, because they got advice and they got it in a timely manner. It's a matter of having somebody with them making sure they do it.” (Theme: accountability)
Recommendations for Improvement
• Tailor the program to address individual needs. For example, improve accessibility for those who are less tech-savvy. (Theme: personalization) • Improve user experience by integrating BMIQ with other platforms and allowing more human interaction. (Theme: integration) • Connect the program to the EHR to enable data sharing and communication. (Themes: integration, communication) • Incorporate cultural diversity in terms of menus and other tools (e.g., availability of other cuisines). (Theme: information/resources, personalization)

In addition, this group cited the educational sessions (i.e., videos and written information) as the most useful feature for patients, followed by the weight and diet tracking tools. For example, one provider stated that:

*Being able to see your weight and see your progress and having those lessons. I heard a lot of good things from patients on that.*

When asked about suggestions or recommendations for improving the online program, some providers suggested tailoring the program to address individual needs and improving accessibility for patients who are less tech-savvy. Some providers suggested connecting the program with other platforms and to the EHR to enable data sharing and communication. Consistent with this notion, some of the most important themes related to their suggestions or recommendations to improve the online program included personalization, integration, communication, and information/resources. Some representative comments from providers are included in Table 3.

#### 3.2.4 Provider attitudes about the support and outreach from population health managers

Most providers stated that the support and outreach from PHMs were an important component of the intervention. Most of the physicians felt that the support and outreach that patients received from the PHMs were notably helpful and had helped patients to manage their weight. They mentioned that patients appreciated the interaction with the PHMs. When PHMs were asked about the value of their support, they highlighted that their contributions were most valued by patients who were more committed to the program compared to those who were less engaged. Below are representative comments from providers:

*For the ones that did (feel they needed help) it actually worked because it helped them to stay accountable.*

*With some patients it was helpful for them because hearing someone cheering them up helped. (With others) they felt a little bit uncomfortable about what they were doing.*

In terms of the challenges related to the support and outreach component of the intervention, PHMs felt that at times that they could not address patients’ questions and needed to reach out to other providers to troubleshoot (e.g., when patients requested advanced nutritional support). One PHM also mentioned that it was hard to connect with patients throughout the study:

*People are busy, and they've got a lot of things coming at them and trying to either text them or email them or phone them. It's hard to connect.*

Specific recommendations to improve the support and outreach component of the study included conducting outreach based on the preference and needs of the patients (e.g., timing, frequency, and content). Some providers also stated that there is a need to increase awareness about outreach and support as well as involve other healthcare providers in the support (e.g., dietitians).

#### 3.2.5 Providers’ desire to use program in the future

Overall, most of the providers stated that they would like to be involved with this type of intervention in the future. For example, one physician stated:

*I would definitely recommend the (hospital) adopt some kind of online weight loss program and of course if they paired it with a person or coaches, that would be great. I think that would be a huge assistance for people.*

The majority of providers recommended that patients use online weight management programs and thought that healthcare institutions should consider adopting and promoting online weight management programs, like the one used in this study.

## 4 Discussion and conclusion

### 4.1 Discussion

In this qualitative study, we conducted semi-structured interviews with primary care patients and providers who were involved in the PROPS Study, to obtain more detailed feedback about the interventions and to help understand the results from the trial. Our findings suggest that both patients and providers were satisfied with the interventions. Some of the important themes about the interventions that were identified from both patient and provider interviews include information/resources, time/efficiency, integration, communication, and personalization. Most patients felt the online program was helpful and easy to use, although some would have liked it to be more personalized. Patients in the combined intervention group reported that the additional support and outreach from the PHMs were critical for their success, as they provided accountability. This is consistent with the results from the trial, which showed that weight loss at 12 months was greater among patients in the combined intervention group than those in the online only group. Some patients reported they would have liked more involvement from their physician or from a dietitian.

Providers also had positive attitudes about the interventions and felt that they improved the quality of care for their patients, although some had few comments about the online program because they were minimally involved. Similar to what patients said, some providers suggested that the information could have been better tailored to individual patients’ needs. Providers also attributed patients’ success in part to the direct support and outreach from the PHMs.

The results from the PROPS Study are consistent with prior studies showing that online weight management programs can be effective for helping people achieve and maintain weight loss, including in the primary care setting [[24], [25], [26]]. To our knowledge, this was the first study to demonstrate that an online program can be integrated with existing population health management support delivered by nonclinical staff and implemented in routine primary care. Other qualitative studies have had similar findings as those reported here, suggesting that patients and providers are satisfied with online programs and feel that they should be more widely used in the future, with some areas for improvement [[27], [28], [29]]. For example, one study that included semi-structured interviews with 20 primary care patients reported that although patients were initially motivated to use online programs, barriers to continued use included time and effort, negative reactions to prompts and reminders, and beliefs that the information being conveyed was not useful [29]. Participants recommended that online programs should be personally tailored whenever possible, to allow for choice of style, format, and number of reminders. In addition, this study recommended that the use of face-to-face support combined with online support may be helpful when implementing online programs in primary care settings. Another mixed methods evaluation indicated that brief human support was important to engagement with the online program and successful weight loss [30].

The results from our study complement and extend the results from the PROPS trial and highlight the potential value of online weight management programs and population health management support in primary care settings. The findings show that the online program combined with population health management support was effective, and that both primary care patients and providers were satisfied with the interventions. The implications are that the combined intervention, including the online program plus population health management support, should be implemented on a broader scale in the primary care setting in the future, with more tailoring of information to meet patients’ needs.

This study has several limitations. The study was conducted at a single institution and only among primary care patients; therefore, the results may not be generalizable to other settings. Furthermore, we restricted the interviews to patients in the OP and CI groups who had completed the 18-month follow-up survey and to providers who had at least six patients enrolled in the trial; this sampling method excludes patients who had low engagement and providers who had fewer patients. Thus, patients and providers who participated may not be representative of all patients and providers in the trial and their subjective experience with the interventions. However, we wanted to focus on those who had been most engaged in the study, in order to get more detailed feedback about the interventions that would be helpful for future improvements.

### 4.2 Innovation

To our knowledge, this is the first study to utilize an online weight management program combined with population health management support to address overweight and obesity in the primary care setting. We previously found that this innovative approach was more effective for weight loss compared to the online weight management program alone or to usual care, and the findings from this qualitative sub-study give additional information about patients’ and providers’ attitudes about and experience with the interventions. This information can be used to improve and expand on these interventions in the future.

### 4.3 Conclusion

In summary, both primary care patients and providers had positive feelings about the interventions in the PROPS Study. In particular, they felt that the additional support and outreach from the population health managers, in conjunction with the online program, was critical to the success of the interventions. Institutions should consider implementing these interventions, including an online program combined with population health management support, on a broader scale in the primary care setting, with more tailoring of information to patients’ needs.

## Declaration of Competing Interest

Dr. Bates consults for EarlySense, which makes patient safety monitoring systems. He receives cash compensation from CDI (Negev), Ltd, which is a not-for-profit incubator for health IT startups. He receives equity from ValeraHealth which makes software to help patients with chronic diseases. He receives equity from Clew which makes software to support clinical decision-making in intensive care. He receives equity from MDClone which takes clinical data and produces deidentified versions of it. He will be receiving research funding from IBM Watson Health. Dr. Rozenblum reports having an equity in Hospitech Respiration Ltd, which makes Airway Management Solutions. He is also receiving research funding from IBM, Boston Scientific Corporation, and MedAware. Dr. Halperin receives cash compensation and equity from Form Health, Inc.

## Appendix A Supplementary data

Supplementary material 1 Patient Interview Guide Supplementary material 1

Supplementary material 2 Provider Interview Guide Supplementary material 2
