# Supplementary material for: Primary care patients’ and providers’ perspectives about an online weight management program integrated with population health management: Post-intervention qualitative results from the PROPS study
Source: PEC Innov. 2022 Jun 11;1:100057. doi: 10.1016/j.pecinn.2022.100057 (PMC10194385; doi:10.1016/j.pecinn.2022.100057)
Supplement: Supplementary material 1 — Patient Interview Guide [file mmc1.docx]

**Appendix A: Patient Interview Guide**

**Interview Guide**

(Version for Online Program & Combined Intervention patients)

**Interview Logistics**

| **Interview Date**  (month/day/year) |  |
| --- | --- |
| **Interviewer** |  |
| **Length of Interview** (minutes) |  |
| **Additional Notes** |  |

**INTERVIEW QUESTIONS**

| **Your activities related to the BMIQ online program:**   1. We would like to know how you accessed the BMIQ online program (desktop computer, laptop, tablet, or smartphone)? Why? How was it for you? 2. During the study period, how often did you log in to the BMIQ online program (e.g., how many times per day, week, or month)?   **PROBE:** How was it used initially and over time? |
| --- |
| **Your experience with the BMIQ online program**   1. In general, what was your experience with the BMIQ website?      1. How did you feel about the look of the program? how easy was it for you to move through the various features/tools?   **PROBE:** Was it user-friendly? Did you have any technical difficulties? Please describe.  **PROBE:** Do you have any other suggestions or comments for us about the layout of the program?   1. Overall, to what extent did you find the BMIQ online program useful?   **PROBE:** to what extent do you feel that using the BMIQ online program has helped you to manage your weight? and how?   1. Specifically, what would you consider the most useful functions/features in the BMIQ online program? What tools are most beneficial?   **PROBE:** Did you find the:   - Sessions useful (written information & video)? Why or why not? - Tools for tracking food, physical activity, and weight useful? Why or why not? - Electronic messages/reminders? Why or why not?  1. What are the main weaknesses and/or challenges of using the BMIQ online program? 2. Do you have any other suggestions or recommendations for how we can modify or improve the BMIQ online program? |
| ***Combined Intervention (CI) patients only***  **Your experience with the support and outreach that you have received from your primary care practice related to the online program (BMIQ).**     1. Overall, what was your experience with the support and outreach that you received from your primary care practice related to the BMIQ online program?   **PROBE:** ask specifically about the population health manager and primary care provider, dietitian/nutritionist   1. Did you find this support and outreach useful? 2. Do you have any other suggestions or recommendations for how we can modify or improve the support and outreach related to this program? |
| **Study & program implementation and management:**   1. In general, how was your experience in the study with regards to content support and/or technical support?   **PROBE:** Did you use any support during the study?  **PROBE:** Did you know who to contact with questions or concerns? |
| **DESIRE TO USE PROGRAM IN THE FUTURE**   1. Now that you’ve completed your participation in the PROPS Study, do you think you will continue to use BMIQ? Why or why not?   **PROBE (*CI pts only)***: Now that you have used the BMIQ online program and received weight-related support and outreach from your primary care practice, do you think you will continue to use BMIQ? Why or why not?  **PROBE:** Recommend to other friends and family members? |
| 1. In general, what other suggestions or comments might you have for us? |

Thank you for participating. Your opinions and input are very much appreciated and will help us to learn more about this intervention and hopefully improve it.

**THANK YOU VERY MUCH**
