# Supplementary material for: Primary care patients’ and providers’ perspectives about an online weight management program integrated with population health management: Post-intervention qualitative results from the PROPS study
Source: PEC Innov. 2022 Jun 11;1:100057. doi: 10.1016/j.pecinn.2022.100057 (PMC10194385; doi:10.1016/j.pecinn.2022.100057)
Supplement: Supplementary material 2 — Provider Interview Guide [file mmc2.docx]

**Appendix B: Provider Interview Guide**

**Key Informant Interview Guide:**

**Interview Logistics**

| **Interview Date**  (month/day/year) |  |
| --- | --- |
| **Interviewer** |  |
| **Length of Interview** (minutes) |  |
| **Additional Notes** |  |

**INTERVIEW QUESTIONS**

| **General information about your role and involvement in the PROPS study**   1. How would you describe your main role at Brigham and Women’s Hospital? 2. During the study period, were you involved in the PROPS study? If yes, in which arm and how?   **PROBE:** The stand-alone online weight management program (BMIQ), or the combined intervention (online weight management program plus population health management support)   1. During the study period, did you receive any information about the status of your patients related to their participation in the study? If yes, what type of information? and what did you do with this information? 2. Did you talk with patients about their participation in the study?   **PROBE:** Did you talk with them about their experience, progress, difficulties? |
| --- |
| **General attitude towards the PROPS Study**   1. Now I would like to focus on your general attitude towards the PROPS Study. What is your overall impression of the study? 2. I’d also like to know more about your impressions of how the study has affected patient care. To what extent do you feel that the study has helped   you as a [clinician / stakeholder] to provide better care for your patients? and  how?   - 1. Do any examples stand out to you as particularly interesting?   2. Has the study impacted patient care in any ways that you didn’t anticipate? |
| ***For providers familiar with BMIQ***  **Your attitudes related to the BMIQ online program**   1. What do you think about the BMIQ online program that was tested in this study? 2. To what extent do you feel that using the BMIQ online program has helped your patients to manage their weight? and how? 3. Specifically, what would you consider the most useful functions/features in the BMIQ online program? What tools have been most beneficial?   **PROBE:**   - Sessions (written information & videos about certain topics, such as nutrition, physical activity, goal setting, etc.) - Calorie goals and meal plans - Tools for tracking food, physical activity, and weight - Electronic messages/reminders? - Professional interface (summary page, progress notes, reports)  1. What are the main weaknesses and/or challenges of using an online   program like BMIQ?   1. Do you have any other suggestions or recommendations for how we could modify or improve the BMIQ online program? |
| ***For providers with patients in the Combined Intervention (CI) group***  **Your attitudes related to the combined intervention (BMIQ online program & PHM outreach related to weight management)**   1. In general, what do you think about the combined intervention arm in this study: BMIQ online program & outreach from population health managers or other providers? 2. To what extent do you feel that the support and outreach that patients received from the population health managers (or any other provider) related to the BMIQ online program has helped them manage their weight? and how? 3. To what extent do you feel that the support and outreach that your patients received has helped you as a [clinician / stakeholder] to provide better care for your patients? and how? 4. What are the main weaknesses and/or challenges of the support and outreach that patients received during this study? 5. Do you have any other suggestions or recommendations for how we can modify or improve the support and outreach related to this program? |
| **Desire to use program in the future**   1. In the future, would you like to be involved with this type of intervention? If yes, how? 2. Would you recommend patients use online weight management programs, like BMIQ? Why or why not? 3. Do you think that institutions like yours, should adopt and promote online weight management programs, like BMIQ? |
| 1. In general, what other suggestions or comments might you have for us? |

Thank you for participating. Your opinions and input are very much appreciated and will help us to learn more about this intervention, its potential effect and hopefully improve it.

**THANK YOU VERY MUCH**
